# Is surgery without curettage effective for periacetabular Metastasis? Insights from a survival study of 93 patients

**DOI:** 10.1016/j.jbo.2024.100643

**Published:** 2024-10-10

**Authors:** Thomas Amouyel, Marie-Hélène Vieillard, Alain Duhamel, Carlos Maynou, Martine Duterque-Coquillaud, Cyrielle Dumont

**Affiliations:** a Univ. Lille, CNRS, INSERM, CHU Lille, Institut Pasteur de Lille, UMR9020-U1277 -CANTHER - Cancer Heterogeneity Plasticity and Resistance to Therapies, F-59000 Lille, France; b CHU Lille, Service d’Orthopédie 1, F-59000 Lille, France; c CHU Lille, Rheumatology Department, F-59000 Lille, France; d Oscar Lambret Center – CISSPO, 3 rue Frédéric Combemale – BP 307, F-59020 Lille Cedex, France; e Univ. Lille, CHU Lille, ULR 2694 - METRICS : Évaluation des technologies de santé et des pratiques médicales, F-59000 Lille, France; f CHU Lille, Département de Biostatistiques, F-59000 Lille, France

**Keywords:** Acetabular, Bone metastasis, Survival, Total hip arthroplasty, Curettage

## Abstract

**Background:**

The main aim of this study was to analyse the 6-month survival rates in *peri*-acetabular metastasis patients undergoing total hip arthroplasty (THA) with an acetabular cage and without curettage. The secondary objectives were to analyse the global survival rates, the factors influencing patient survival and to evaluate mechanical complication rates after THA.

**Methods:**

This study was carried out on a cohort of 93 consecutive patients who underwent THA with an acetabular cage without curettage for acetabular metastasis or multiple myeloma lesions between 2010 and 2020. The National Death Registry was consulted to obtain the exact date of death of the patients; the minimum follow-up time was 2 years.

**Results:**

The 6-month survival rate for all types of cancer was 78 % [68 – 85], the 1-year survival rate was 66 % [55 – 74], and the 5-year survival rate was 26 % [17 – 36]. The median overall survival for the cohort was 24.37 months [16.10 – 32.63]. The mean overall survival was 46.02 months [32.89 – 59.16]. At last contact, 86 % of the operated patients were walking again.

No patient died from surgery. The ECOG performance status score, the number of bone metastatic sites, the presence of visceral metastases and the number of lines of systemic therapy undertaken prior to surgery were negative survival factors. Three patients (3.2 %) had early prosthetic dislocation, 2 patients (2.2 %) showed aseptic loosening of her partial hip implant after 10 and 11 years respectively and 4 patients (4.3 %) had an early infection treated by debridement, antibiotics and implant retention to control the infection. During the follow-up period, no new femoral metastases were detected in any patient.

**Conclusion:**

Surgery without curettage is an effective treatment for periacetabular metastasis. It gives reliable results, regardless of the type of acetabular lesion, allowing most patients to walk again and does not modify the patient’s survival.

## Highlights

• Survival rate of operated metastatic acetabular lesions exceeds 2 years.
• Surgery of metastatic acetabular lesions allows most patients to walk again.
• Multiple bone metastases and visceral metastases have negative impact on survival.
• High ECOG score and multiple lines of systemic therapy impact negatively survival.

## 1 Introduction

After the spine, the pelvis is one of the most common sites for bone metastases.[1] Although iliac crest, pubis and ischium metastases can be treated with non-operative therapies such as cementoplasty, radiotherapy, thermotherapy or percutaneous screw fixation, most *peri*-acetabular metastasis cases are at risk of mechanical failure and thus require a total hip arthroplasty (THA).[2], [3], [4], [5], [6], [7] Although a standard femoral stem can be used, acetabular component fixation requires the use of cement-and-pin augmentation or antiprotrusio cages to ensure stable reconstruction through a more extensive surgical approach [8], [9].

The main objective of surgery is to improve the quality of life of the patient, by decreasing pain and allowing full weight-bearing, without reducing life expectancy [10]. There are several acetabular reconstruction techniques, but all the clinical studies focus on implant survival rather than on the impact of the surgery on the overall patient survival rate: modified Harrington procedures use metal pins and cement to fill the metastatic lesion, thereby allowing the use of a standard cemented cup, or acetabular reinforcement devices with or without metastasis curettage [11], [12], [13], [14], [15], [16], [17], [18]. These procedures are challenging for the surgeon, who has to deal with large and complex acetabular reconstruction, leading to increased surgery time, blood loss and risk of complications leading to readmission [19].

Most of the time, only one type of procedure is performed by a surgical team, we use dual-mobility implants cemented in a reinforced antiprotrusio cage without curettage of the metastasis for acetabular metastasis associated with long femoral stem to shorten the surgery and reduce the number of complications [20].

The primary objective of this study was to analyse the 6-month survival rates of patients operated for *peri*-acetabular metastasis. The secondary objectives were to analyse the overall survival rates, to analyse the factors influencing patient survival and to evaluate mechanical complications after THA.

## 2 Material and Methods

### 2.1 Study design

We conducted an exploratory monocentric, retrospective study. We included all adult patients operated on at our university hospital for THA with acetabular metastasis or multiple myeloma acetabular lesions between 2010 and 2020. Patients were selected from the surgical register based on the implant list. We excluded patients with only femoral metastases, patients with primary lesions of the acetabulum, patients without metastatic lesions, and patients who underwent revision surgery. We included 93 patients, the main objective of the surgery was to enable the patients to stand upright without the risk of fracture. All patients had a minimum follow-up of 2 years or until death. Data were collected and anonymized from the patient’s hospital record.

### 2.2 Data collection

Age, BMI, sex and performance status were collected from the patient's file, and all the oncological diagnoses were confirmed by biopsy prior to surgery. The number of bone, spinal and visceral metastases were evaluated on a PET scan. Data on the number of lines of systemic treatment before the discovery of the acetabular metastasis was extracted, a Katagiri score was calculated, and presentation at a multidisciplinary team meeting (MTM) was noted [21]. The ECOG performance score was calculated, and age at initial cancer diagnosis, age at surgery, symptomatic nature of the lesion and presence of a femoral lesion concomitant to the acetabular lesion were also recorded. Acetabular lesions were classified according to the Harrington classification [8].

The National Death Registry was consulted during the final analysis of the data to obtain the exact date of death of the patient, if necessary.

The study followed the MR 004 reference methodology and obtained approval from the French national data protection authority (CNIL No. DEC21-268).

### 2.3 Surgical procedure

Pre-operative embolization was performed the day before surgery for kidney cancer metastases and other cancers with high bleeding potential.

**Fig. 1:**
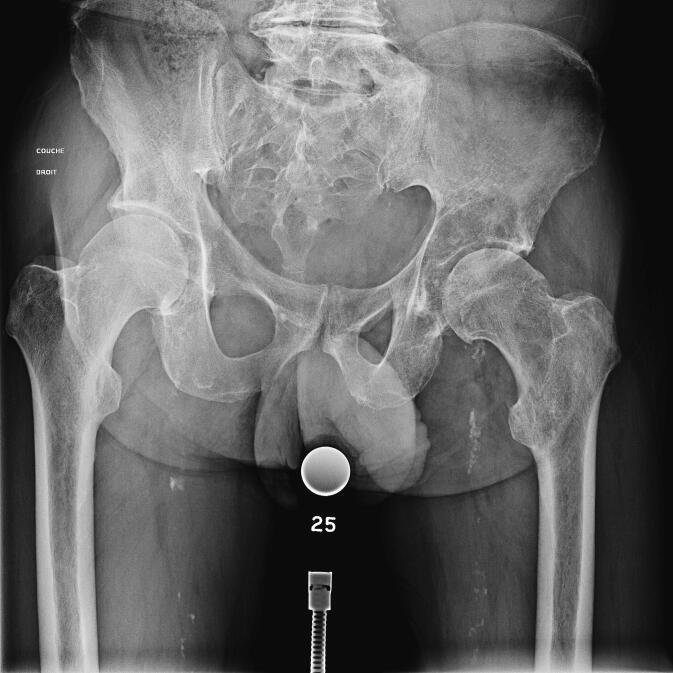

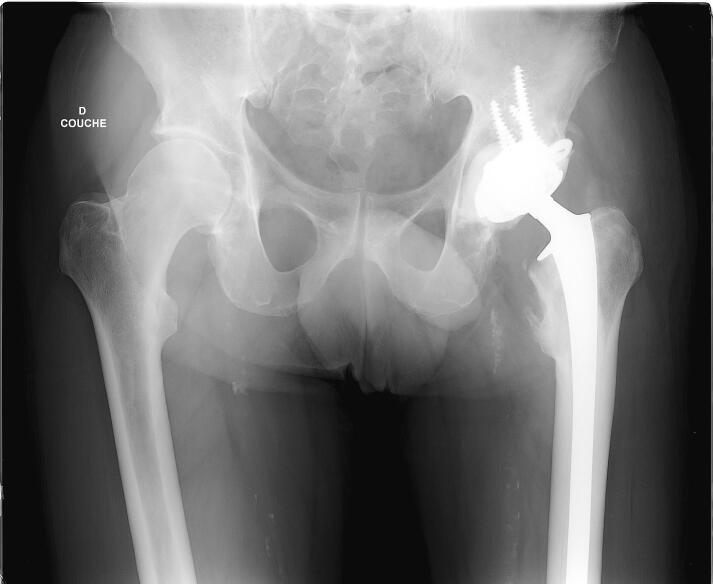
A: pre-operative radiograph of a 61-year-old man with lung cancer involving metastasis in the acetabulum. B: Post-operative radiograph. The patient underwent total hip arthroplasty with a Müller reinforcement ring.

**Fig. 2:**
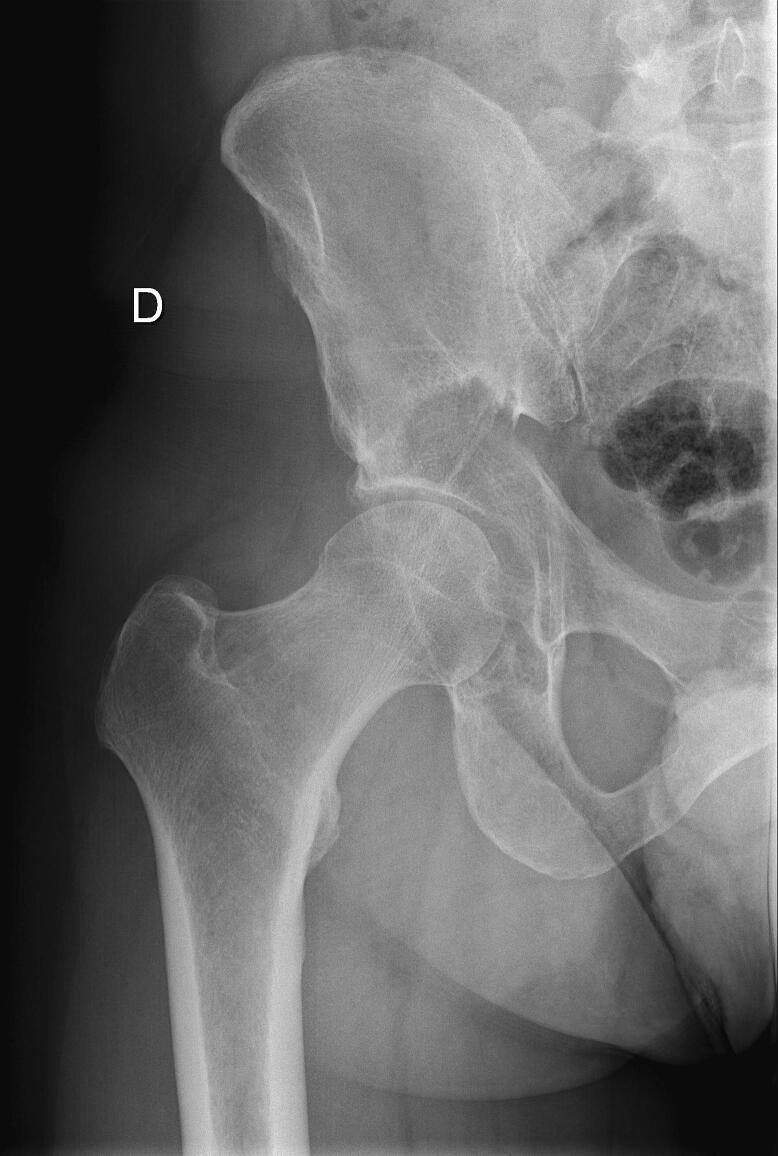

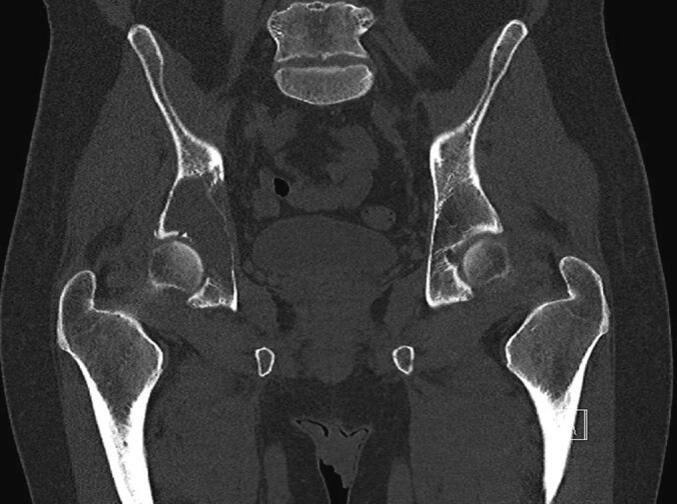

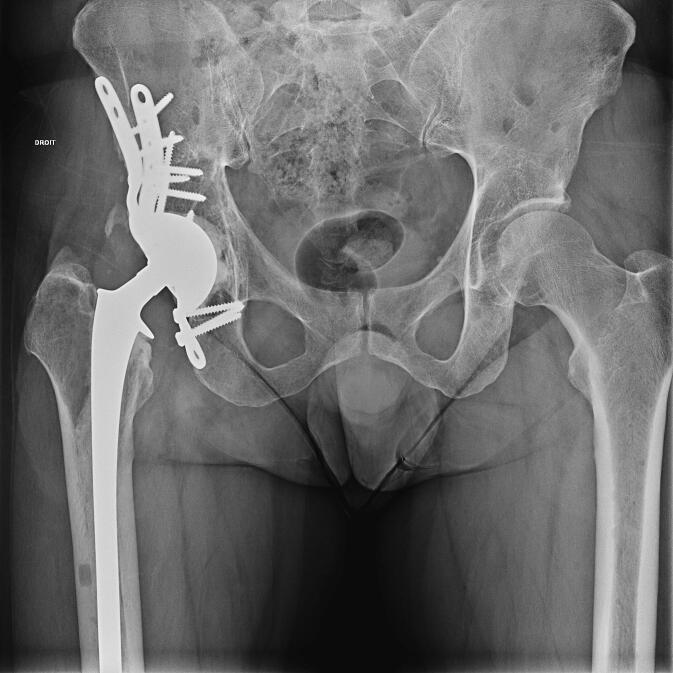
A pre-operative radiograph of a 46-year-old man with a multiple myeloma involving the acetabulum. B: pre-operative CT scan. C: Post-operative radiograph. The patient had a total hip arthroplasty with a partial pelvis implant.

All patients underwent surgery under general anaesthesia, positioned in lateral decubitus. All patients underwent hip arthroplasty with a cemented long stem (Lubinus SP2; Waldemar Link, Hamburg, Germany) and acetabular reinforcement depending on the size of the lesion (Müller® acetabular reinforcement ring (Zimmer, Winterthur, Switzerland) or LINK® Endo-Model® partial pelvis replacement prosthesis (Waldemar Link, Hamburg, Germany)) (Fig. 1, Fig. 2) using an extended posterior approach. A POLARCUP Dual-Mobility cup (Smith & Nephew Orthopaedics AG, Rotkreuz, Switzerland) was systematically cemented into the acetabular reinforcement with Palacos®R + G cement (Heraeus Medical GmbH, Wehrheim, Germany). No lesions were curetted, and all patients received post-operative radiotherapy 4–6 weeks after surgery once the wound was completely healed. Resumption of systemic therapy was left to the discretion of the patient's oncologist once healing was complete. Initiation or continuation of bone target therapy was discussed in MTMs.

Full weight-bearing was allowed immediately after surgery, depending on the risk of fractures due to other metastatic lesions. Patients were reviewed at 1 month, 3 months, 6 months and then annually for clinical and radiographic assessments by their surgeon.

### 2.4 Statistical analysis

Statistical analysis was performed using SPSS software (version 26.0, SPSS Inc./IBM, Chicago, IL, USA) and R version 4.0.3 with EZR v1.54. Quantitative variables were expressed as means (standard deviation) for normal distribution or medians (interquartile range, IQR) otherwise. Normality was assessed using histograms and the Shapiro-Wilk test. Categorical variables were expressed as numbers (percentage). Median survival data are given with their 95 % confidence intervals (CI). Survival was calculated using a univariate Cox method and the survival curve was constructed using the Kaplan-Meier estimate and 95 % CI. Univariate Cox models were used to calculate the risk factors’ hazard ratios with their 95 % CI, after checking for proportional hazards and checking the log-linearity assumption with martingale residuals. Considering the small number of events, we were unable to carry out a multivariate analysis. The significance level was set at 0.05.

## 3 Results

Ninety-three patients were included, their demographic data are given in Table 1.

**Table 1:** Demographic data (N = 93). ENT: Ear Nose Throat, Other: Bladder, cholangiocarcinoma, colorectal, endometrial, gastric, liver, lymphoma, metastatic sarcoma, melanoma, uterine.

	n = 93
Mean age (yr)	60 (σ = 12)
Mean BMI	25.22 (σ = 5.9)
Sex (No. of patients)	
Male	36 (39 %)
Female	57 (61 %)
Cancer subtype (No. of patients)	
Breast	45 (48 %)
Multiple myeloma	10 (11 %)
Lung	9 (10 %)
Prostate	8 (9 %)
Renal	6(6 %)
ENT	3 (3 %)
Thyroid	2 (2 %)
Other	10 (11 %)

Seventy-four patients were discussed in an MTM before surgery. The mean time between MTM and the surgical procedure was 43 days (σ = 27.5 days). The mean number of other metastatic lesions was 3.76 (σ = 1.9), the mean Katagiri score was 3 (σ = 1.8), the metastasis revealed the primary cancer in 43 patients, 29 patients had visceral metastases and 66 spinal metastases.

Seventeen patients were ECOG-0, 47 were ECOG-1, 23 were ECOG-2, 5 were ECOG-3 and only one was ECOG-4. Acetabular lesions were analysed according to the Harrington classification: 27 were in Class 1 (24 with femoral lesion), 17 Class II (10 with femoral lesion) and 49 Class III (16 with femoral lesion) [8].

The mean operative time (from incision to bandage placement) was 126 min [69–200]. For Müller acetabular reinforcement ring, the mean operative time was 118 min [75–154], and 143 min [69–200] for LINK® Endo-Model® partial pelvis replacement prosthesis.

At last contact, 86 % of the operated patients were walking again with or without a cane.

### 3.1 Survival analysis

The 6-month survival rate for all types of cancer was 78 % [68 % − 85 %], the 1-year survival rate was 66 % [55 % − 74 %], and the 5-year survival rate was only 26 % [17 % – 36 %].

**Fig. 3:**
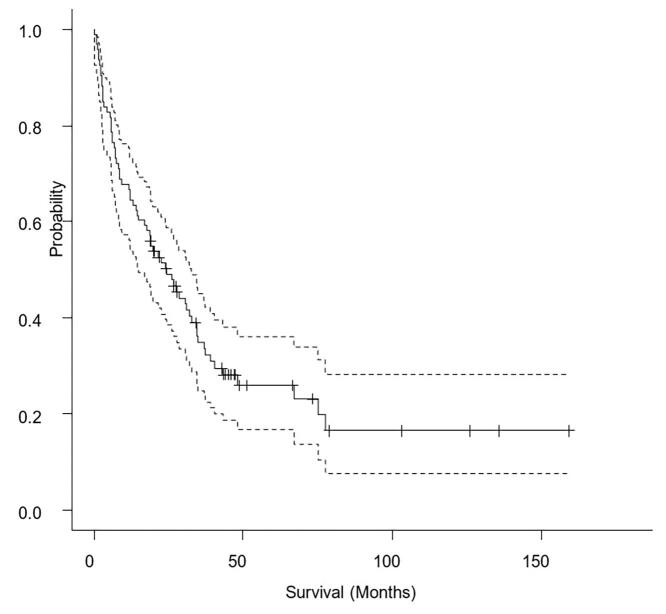
Overall survival curve (solid line) with 95% confidence interval (dashed lines).

The median overall survival for the series was 24.37 months 95 % CI [16.10–––32.63] (Fig. 3), median survival by cancer type is shown in Table 2. The mean overall survival was 46.02 months [32.89–––59.16].

**Table 2:** Median survival by cancer type (if involving 3 or more patients), ENT: Ear Nose Throat.

Cancer Type (n = )	Median survival time (months) [min – max]
Myeloma (10)	41.05 [2.93 – 79.10]
Prostate (8)	32.93 [1.63 – 67.27]
Breast (45)	26.13 [1.80 – 159.50]
Kidney (6)	10.97 [2.47 – 28.50]
ENT (3)	9.33 [1.13 – 44.10]
Lung (9)	5.87 [0.23 – 49.03]

The univariate hazard ratios of the variables analysed in this study are shown in Table 3. We choose ECOG Stage 0 and Class I of the Harrington classification as the reference modalities.

**Table 3:** Univariate hazard ratio of factors influencing survival at the significance level of 0.05 (*).

Variable	Hazard ratio	95 % CI	p
Full weight-bearing	0.073	0.035 – 0.152	0.001*
Cancer discovered with the metastatic lesion	0.796	0.489 – 1.294	0.357
Post-operative radiotherapy	0.885	0.482 – 1.624	0.693
Spinal metastasis	0.945	0.542 – 1.649	0.843
BMI	0.990	0.946 – 1.037	0.677
Time from MTM to surgery (weeks)	0.996	0.985 – 1.007	0.450
Age at cancer diagnosis (years)	1.005	0.989 – 1.022	0.540
Age at time of surgery (years)	1.007	0.989 – 1.026	0.439
Femoral lesion	1.113	0.687 – 1.804	0.664
Symptomatic lesion	1.173	0.597 – 2.305	0.643
Number of lines of cancer therapy	1.209	1.021 – 1.430	0.027*
Presentation in MTM	1.241	0.673 – 2.289	0.489
Number of bone metastatic sites	1.264	1.098 – 1.455	0.001*
New metastasis from a known cancer	1.427	0.858 – 2.373	0.170

Katagiri score	1.581	1.358 – 1.839	0.001*

Visceral metastasis	2.529	1.526 – 4.192	0.001*

ECOG (overall) Stage 0 Stage 1 Stage 2 Stage 3–4	Reference 2.634 2.745 5.768	1.168 – 5.939 1.149 – 6.562 1.819 – 18.300	0.023* 0.020* 0.023* 0.013*

Harrington classification (overall) Class I Class II Class III	Reference 1.998 0.835	1.014 – 3.938 0.476 – 1.465	0.024* 0.046* 0.528

The type of cancer was analysed; however, the proportional hazards hypothesis was not validated, preventing analysis of the hazard ratio in this case. The number of bone metastases, the number of lines of previous systemic treatment, the presence of visceral metastases, a higher ECOG score and a higher Harrington classification were significant negative survival factors.

### 3.2 Complications

Three patients (3.2 %) had early prosthetic dislocation, reduced under general anaesthesia with no subsequent episodes or reoperation.

One patient (1.1 %) showed aseptic loosening of her partial hip implant after 10 years, she underwent a two-stage THA with a custom-made implant designed from a CT scan; a second patient (1.1 %) experienced a loosening of her implant after 11 years, but due to the relatively good tolerance to the induced pain, the age of the patient and the risks of revision, she was not re-operated.

Four patients (4.3 %) had an early infection treated by debridement, antibiotics and implant retention to control the infection. However, these patients did not undergo post-operative radiotherapy and could not resume chemotherapy. The two patients with hormone-dependent breast cancer metastases were able to resume their hormone therapy with lifetime suppressive antibiotic therapy. The other two patients (one bladder cancer and one lung cancer) died early due to local and systemic metastatic progression and the inability to resume systemic therapy. The median survival for infected patients was 13.20 months 95 % CI [0.00 – 29.14] and the mean survival was 17.16 months [4.91–29.44].

Three operated patients were discussed in the MTM after surgery. One patient with lung cancer was re-irradiated one year after surgery and conventional post-operative radiotherapy. Another patient with breast cancer was re-irradiated four years after surgery. The third patient, who had myeloma, underwent cementoplasty for her progressive acetabular lesion. This was done three years after surgery.

During the follow-up period, no new femoral metastases were detected in any patient.

## 4 Discussion

Acetabular metastases are technically challenging lesions to manage, requiring extensive surgery on patients weakened by metastatic disease and by the burden of adjuvant treatments such as chemotherapy or radiotherapy sessions.

The 6-month survival rate of patients in our cohort of THA with acetabular support rings and without curettage of the lesion was 78 %, with a median survival of 24.37 months [16.10 – 32.63] for all types of cancer combined. Our results are consistent with recent studies on the management of acetabular metastases. Plaud *et al.* reported a 6-month survival rate of 76.2 % in a similar population of 21 patients who underwent a Harrington procedure with THA between 2005 and 2020, as did Tsagozis *et al.*, with median survival rate of 12 [7], [8], [9], [10], [11], [12], [13], [14], [15], [16] months in 70 patients, albeit with a 5-year survival rate of only 7 % [22], [14]. Tillman *et al.* studied longer-term survival by including metastatic patients operated between 1996 and 2018, with a 5-year survival of 33 %, which is slightly higher than our cohort (26 %) [11]. However, these results differ from the study by Kask *et al.* which found a median survival of 8 months, with 14 out of 89 patients surviving less than 2 months after surgery, despite having the same inclusion criteria [23].

These similarities, despite the lack of metastatic curettage in our cohort, suggest that curettage does not improve survival. In their cohort, Wegrzyn *et al.* curetted the lesions before cementing them and inserted an implant without performing a Harrington procedure. The authors did not report an overall survival rate, only the average survival rates with (18 ± 15 months) and without (39 ± 21 months) visceral metastasis. Those results suggest that there is not any clear benefit to removing the metastasis, given that our average survival rates were 17 ± 3 months and 59 ± 9 months, with and without visceral metastasis, respectively [16].

In addition, curettage does not seem to improve the durability of the implants. In the case of very large defects (Harrington Class III), we used implants seated on the iliac wing and ischium (partial pelvis implants) to maximize bone fixation and the primary stability of the armature, leading to only two implants loosening after more than 10 years following surgery. In the case of Harrington acetabular reconstruction, the revision rates for loosening typically vary from 0 to 13 % in studies with small populations [11], [22], [24], [25], [26], [27].

Hip function was restored with early weight-bearing in 86 % of our patients. Those who could not stand upright were impaired by other lesions, especially spinal lesions, or were in a general condition that did not allow mobilization, in which case the prosthesis only served to reduce pain during transfers. Full weight-bearing occurs in all reported cohorts, whether with a modified Harrington reconstruction technique or with an acetabular reinforcement system [11], [16], [22], [28], [29].

We analysed the pre-operative predictive survival factors. Unsurprisingly, non-zero ECOG scores were a significant risk factor for death, as well as the type of cancer. However, given the predominance of breast cancer and the small numbers of patients with other types of cancer, it was not possible to rank them separately. These results are in accordance with the analysis carried out by Baumber *et al.* who, in a retrospective analysis of 164 metastatic patients, found high ASA scores, hyperleukocytosis, hyponatremia, tachycardia and type of cancer to be negative factors for survival [30].

Our specific univariate analysis of *peri*-acetabular lesions found the number of bone metastatic sites, the presence of visceral metastases and the number of lines of systemic therapy undertaken prior to surgery to be negative survival factors. These items are found in the Katagiri score as “presence of visceral metastases”, “multiple bone metastases”, “prior chemotherapy” [21]. This score, discussed during multidisciplinary consultation meetings (MTMs), is used to estimate the patient's life expectancy according to various criteria, especially the type of cancer, and thus to select patients for more intensive treatments.

The validity of the Katagiri score to help forecast the benefits of acetabular surgery can be controversial, because the analyses were carried out on a population of 958 metastatic patients, among whom only 7 % underwent surgery and only 2 % were affected with a *peri*-acetabular lesion. This score therefore does not take into account the possible higher mortality risk of performing an extensive surgical procedure on very fragile patients. Future studies should compare patients with this type of lesion who have been operated and those who have not, to estimate the consequences of such surgery and to score surgical prognosis, the aim being to avoid reducing life expectancy to below 6 months, the limit set by the British Orthopaedic Oncology Society [31].

The type of lesion according to Harrington's classification was also generally significant in the univariate analysis. However, only Class II showed a significant, twice higher risk of death. Harrington's Class III being non-significant, it is not possible to draw any conclusions on this risk, which is probably higher according to the overall tumour volume and the progress of the metastatic disease. Furthermore, very advanced Stage 3 patients probably do not undergo surgery due to their low life expectancy; our data collection only included patients who were operated on.

The overall complication rate in our cohort was 9.7 %, which is in the low range of similar cohorts: 9 % for Wegrzyn *et al* [16], 11 % for Kask *et al* [23], 22 % for Tilleman *et al*. [11], 23,8% for Plaud *et al* [22] to 33 % for Rajasekaran *et al* [13] and Tsagosis *et al* [14]. Our technique limits the number of operative steps, with the absence of lesion curettage, no counter-incision at the iliac crest to insert rods, and no radiographic imaging. This approach reduces the risk of infection, which constitutes the most common complication in this type of patient and is a major factor of early death following this procedure, reducing the average survival from 46.02 months [32.89–59.16] to 17.16 months [4.91–29.44].

### 4.1 Limits

Our study was retrospective and we were not able to estimate functional scores. Given the oncological context, function was considered to be of secondary importance, with verticalization and pain reduction being the main focus.

However, this study presents one of the largest series of acetabular reconstructions with more than 2 years of follow-up and verification of the dates of death in the national registers.

A multicentre study can increase the sample size and thus allow for a multivariate analysis of the factors influencing survival in these patients.

## 5 Conclusion

Surgery without curettage is an effective treatment for periacetabular metastasis.

The 6-month survival rate of patients operated on for a metastatic *peri*-acetabular lesion was 78 %, the median survival of patients was 24 months. We identified the ECOG status, visceral metastases, and the number of bone metastases as factors that negatively affect survival. Verticalization was possible for 86 % of patients and only two implants loosened after more than 10 years. The management of acetabular lesions by implanting a support cage without curettage of the metastatic lesion gives reliable results, regardless of the type of acetabular lesion.

## CRediT authorship contribution statement

**Thomas Amouyel:** Writing – original draft, Methodology, Data curation, Conceptualization. **Marie-Hélène Vieillard:** Writing – review & editing, Conceptualization. **Alain Duhamel:** Methodology. **Carlos Maynou:** Writing – review & editing. **Martine Duterque-Coquillaud:** Writing – review & editing, Supervision. **Cyrielle Dumont:** Writing – review & editing, Formal analysis.

## Declaration of competing interest

The authors declare that they have no known competing financial interests or personal relationships that could have appeared to influence the work reported in this paper.
